# Grading of macular perfusion in retinal vein occlusion using en-face swept-source optical coherence tomography angiography: a retrospective observational case series

**DOI:** 10.1186/s12886-019-1134-x

**Published:** 2019-06-10

**Authors:** Magdy Moussa, Mahmoud Leila, Amr Saad Bessa, Mohamed Lolah, Mohsen Abou Shousha, Hazem Medhat El Hennawi, Tarek Abdelrazek Hafez

**Affiliations:** 1 Ophthalmology department, Faculty of Medicine, Tanta University, Tanta, Postal Code 31111 Egypt; 2 MEDIC Eye Center, Tanta, Egypt; 3 Retina department, Research Institute of Ophthalmology, Giza, Egypt; 4 Faculty of Medicine, Alexandria University, Alexandria, Egypt

**Keywords:** SS-OCTA, Grading of macular perfusion, SS-OCTA in retinal vein occlusion

## Abstract

**Background:**

To evaluate the efficacy of swept –source optical coherence tomography angiography (SS-OCTA) in grading macular perfusion in retinal vein occlusion.

**Methods:**

Retrospective observational case series including patients with different types of retinal vein occlusion (RVO). SS-OCTA utilizes OCTARA algorithm to examine the retinal vascular plexuses for the presence of morphological signs of ischemia according to a predetermined grading scheme. The findings were compared with fundus fluorescein angiography (FFA), and swept-source optical coherence tomography (SS-OCT) features. Bivariate correlation, coefficient of determination, and crosstabs procedures were used to calculate inter-variable linear correlation, relative contribution of the tested variables, and multivariate association, respectively.

**Results:**

The study included 144 eyes of 138 patients. The most common type of RVO was branch retinal vein occlusion (BRVO) (53%). The superficial capillary plexus (SCP) and the deep capillary plexus (DCP) did not correlate with each other in all parameters tested. Increased central macular thickness (CMT) and disrupted retinal outer layers (DROL) were associated with increased severity of ischemia in DCP. Disorganized retinal inner layers (DRIL) correlated significantly with the presence of perifoveal capillary ischemia in the SCP and the DCP. Macular ischemia on FFA correlated with ischemia in the SCP layer only. Increased CMT, DROL and DRIL on SS-OCT, and SCP and DCP ischemia on SS-OCTA contributed significantly to diminished best-corrected visual acuity (BCVA).

**Conclusion:**

SS-OCTA is more precise in defining the extent and location of maximum ischemic insult following RVO compared to FFA, hence represents a more efficient grader for ischemic damage in the posterior pole. Increased CMT, DRIL, and DROL on SS-OCT, and SCP and DCP ischemia on SS-OCTA are significant predictors of poor visual outcome.

## Background

The traditional approach for evaluation of macular perfusion following retinal vein occlusion (RVO) consisted of assessment of the superficial capillary plexus (SCP) using fundus fluorescein angiography (FFA), which relayed information on the morphological alteration of the perifoveal capillary network due to ischemia. These changes were used as indicators of visual prognosis after RVO episode according to the area of the macula involved [1, 2]. However, evidence from studies on animal models and normal human subjects revealed that the retinal microcirculation targeted in RVO is far more complex to be truly represented by conventional FFA as the sole imaging modality. The reason is that the inner retina is nourished by a complex vascular network composed of superficial, intermediate, and deep capillary plexuses arranged at different axial levels [3–5]. Moreover, the vascular structure of the deep capillary plexus (DCP) that involves direct communication with major veins and the lack of vascular smooth muscles render the DCP most vulnerable to hemodynamic disturbances following RVO and the ensuing hypoperfusion compared to the SCP [6–8]. Hypoperfusion of the DCP could be detrimental to the visual function due to the pivotal role of the DCP in nourishment of the watershed zone that is located between the inner nuclear layer (INL) and the outer plexiform layer (OPL) and that contains neuronal synapses transmitting visual signal from the photoreceptors [9]. Given that conventional FFA lacks the ability of depth-resolved imaging, it cannot provide useful information on the perfusion profile of capillary plexuses located at different axial depths. The reason is that the relatively fast and profuse leakage of fluorescein dye from the choriocapillaris, along with scattering of light from the nerve fiber layer and deeper retinal layers create fuzzy background fluorescence that impedes detailed imaging of the perfusion profile of the DCP. In fact, FFA reveals only the SCP perfusion. These limitations have been elaborated in several studies that focused on imaging the retinal vascular plexuses using optical coherence tomography angiography (OCTA) technology as alternative to conventional FFA [6, 10–13]. Another important limitation of FFA is that it involves intravenous dye injection which renders it an unrealistic and possibly hazardous follow-up imaging modality given the chronic course of RVO and the need for repeated evaluation [14]. The recently - introduced swept-source optical coherence tomography angiography (SS-OCTA) instated major advances in imaging the retinal vascular plexuses in cases of RVO, and particularly defrayed the drawbacks of conventional FFA. Firstly, SS-OCTA relies on motion contrast techniques to detect moving erythrocytes in relation to static tissue to generate images of the retinal vascular plexuses obviating the need for dye injection. Secondly, SS-OCTA is integrated in the swept-source structural optical coherence tomography (SS-OCT). The SS-OCT incorporates a long wavelength (1050 nm) scanning light, reduced sensitivity roll-off feature, and ultra-high-speed image acquisition. These implements enable deeper penetration with minimal light scattering, hence superior axial resolution and segmentation of different retinal layers. The result is generation of ultra-high-definition images of the SCP and the DCP in separate layers non-invasively [5, 15–19]. The aim of the present study is to evaluate the efficacy of SS-OCTA in grading macular perfusion in cases of RVO and compare the findings with SS-OCT and conventional FFA.

## Methods

This is a retrospective observational case series in which we reviewed the clinical data, FFA, SS-OCT, and SS-OCTA images of all consecutive patients diagnosed with different types of RVO in a private practice from September 2016 to March 2018, and compared the findings.

The study included patients with different types of RVO [central retinal vein occlusion (CRVO), hemicentral retinal vein occlusion (HRVO), and branch retinal vein occlusion (BRVO)]. If the occlusion affected one of the major tributaries of the central retinal vein, the condition was designated major BRVO; whereas whenever the occlusion involved a smaller macular tributary, the condition was designated macular BRVO [20]. Patients with major BRVO had to have concomitant macular involvement to be eligible for the study. All patients participating in the study had to have sufficient resolution of retinal hemorrhages to allow for good quality FFA, SS-OCT, and SS-OCTA. Patients were allowed to enter the study regardless of whether or not they received single or multiple therapies for macular edema or neovascularization secondary to RVO, whether anti-vascular endothelial growth factor (anti-VEGF) agents, intravitreal triamcinolone acetonide (IVTA), and/or laser photocoagulation. Patients with concomitant retinal vascular disease known to affect the retinal vascular plexuses as diabetic retinopathy, retinal vasculitis, combined retinal arterial and venous occlusions, and those with media opacities that were dense enough to impede sufficient image quality for reliable interpretation were excluded from the study.

FFA images were obtained using Topcon TRC 50DX fundus camera (Topcon Corporation, Tokyo, Japan). FFA frames were evaluated for retinal capillary non-perfusion (CNP) following an episode of RVO. RVO was designated ischemic whenever FFA revealed CNP area ≥ 5 disc diameters (DD), and ≥ 10 DD for BRVO and for CRVO and HRVO, respectively [21, 22]. The perifoveal capillary arcade was evaluated in the early venous phase of the angiogram for the presence of signs of macular ischemia. These included disruption of the perifoveal continuum of intercapillary plexus, enlargement of inter-capillary pillars, telangiectasia, capillary pruning and rarefaction, and presence of areas of capillary non-perfusion (CNP). The macula was considered ischemic if ischemic changes of the perifoveal capillary arcade were detected in ≥2 quadrants on FFA.

SS-OCT images were acquired using the DRI OCT Triton machine version 10.11 (Topcon Corporation, Tokyo, Japan); whereas SS-OCTA images were acquired using an integrated blood flow detection algorithm; OCTARA (Optical Coherence Tomography Angiography Ratio Analysis). The SS-OCT scans were evaluated for the presence of macular edema. The central macular thickness (CMT) value was obtained from 1.00 mm diameter circle on the ETDRS grid. Morphological alteration in the retinal microstructure were assessed in a 1.00 mm area centered around the fovea for the presence of disorganized retinal inner layers (DRIL), disruption of the retinal outer layers (DROL) causing loss of the continuity of the external limiting membrane, and/or the photoreceptors inner segment/outer segment (IS/OS) layer. For assessment of DRIL and DROL, the B-scan of maximum affection was selected and two lines were extended temporally and nasally from the fovea up to a distance of 1000 μm in each direction, using the DRI OCT Triton linear measurement tool. DRIL and DROL were considered significant if they involved ≥50% of the 1.00 mm area around the fovea.

OCTA acquisition protocol in the macular region consisted of a 6 × 6 mm area or 9 × 9 mm area centered onto the fovea. Whenever higher resolution was needed, a 4.5 × 4.5 mm area, or a 3 × 3 mm area were used. By default, the integrated software (IMAGEnet 6 ophthalmic data system) deploys automated segmentation to delineate the SCP and the DCP. Images of the SCP were obtained using two slabs located between the internal limiting membrane (ILM) with an offset of 2.6 μm and the inner plexiform layer (IPL) with an offset of 15.6 μm, whereas DCP images were obtained using two slabs located between the IPL with an offset of 15.6 μm and the outer plexiform layer (OPL) with an offset of 70.2 μm. In cases of significant disorganization of retinal layers due to cystoid macular edema (CME) or sizeable sub-retinal fluid, the integrated automated segmentation feature failed to detect the correct boundaries of the SCP and the DCP due to posterior displacement of flow signals, and we had to resort to manual adjustment of the segmentation slab. To perform manual segmentation the operator manually places two segmentation lines at sequential depths guided by corresponding SS-OCT images to reveal the maximum extent of the SCP and the DCP as identified by their respective morphological criteria. The SS-OCTA software generates color-coded flow density maps of the SCP and the DCP, each layer separately. In this map, vessel density in a given area is inferred from the decorrelation motion contrast signal provided by SS-OCTA, where high-flow is represented by increased vessel density and vice-versa [23]. Different vessel densities are then given color-codes and numeric percentage values that reflect the percentage area occupied by blood vessels, where bright red color represents areas of highest density, hence high numeric percentage; whereas dark blue represents areas of no detectable vessels, hence low or zero numeric percentage. Intermediate color shades represent variable grades of vessel density [24].

### SS-OCTA interpretation

**Fig. 1:**
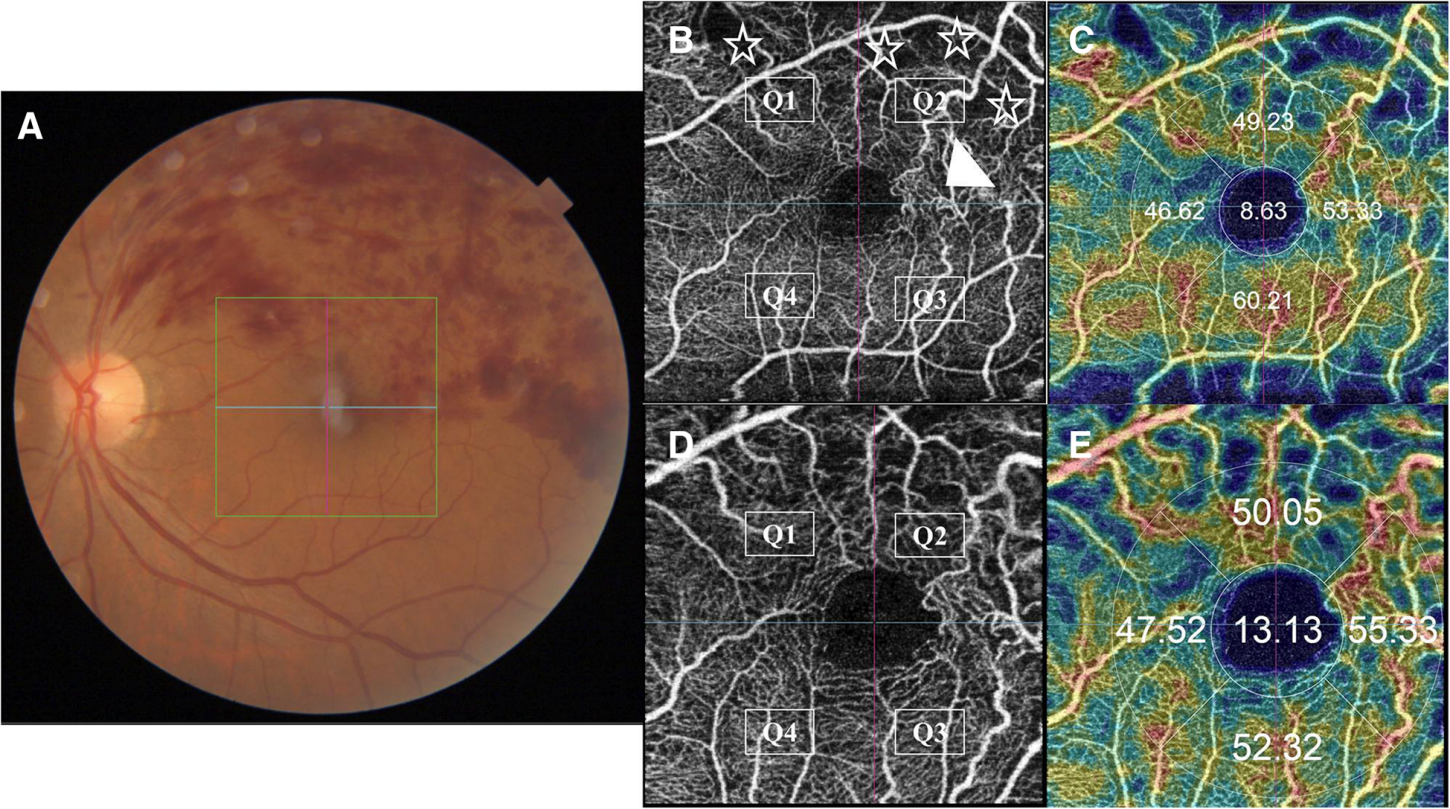
Grading of SCP ischemia. **a**. Color fundus photo of the left eye of a 61-year old male patient with supero-temporal major BRVO. **b**. Corresponding en-face SS-OCTA image of the SCP in a 4.5 × 4.5 mm field. The image is divided by horizontal and vertical crosshairs into 4 quadrants (Q1-Q4). The superior quadrants (Q1-Q2) show flow-voids due to capillary loss (asterisks). Note the dilated shunt vessels straddling the horizontal raphe at the temporal edge of the perifoveal capillary arcade (white arrow-head). **c**. Corresponding flow density map. The percentage area occupied by blood vessels is expressed in numeric values. **d**, **e**. En-face SS-OCTA image of the SCP and the corresponding flow density map displayed in a 3 × 3 mm field for higher definition

**Fig. 2:**
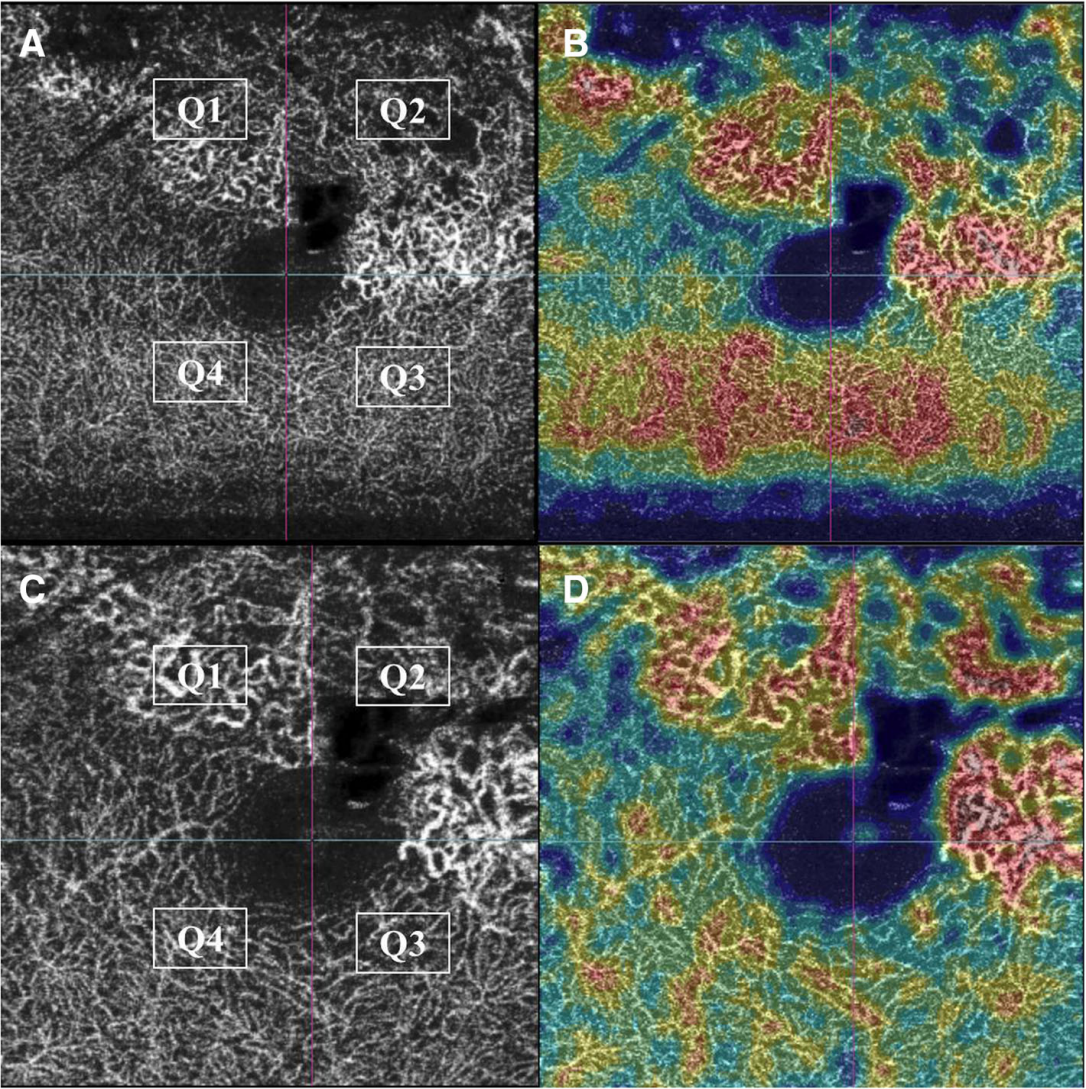
Grading of DCP ischemia. **a**. En-face SS-OCTA image of the DCP in a 4.5 × 4.5 mm field of the same eye of the patient in Fig. 1. Note that the telangiectatic dilatation is more pronounced than that noted on SCP layer. Similarly, cystoid spaces are evident at the supero-temporal aspect of the FAZ that were not detected in the SCP image. **b**. Corresponding flow density map. **c**, **d** En-face SS-OCTA image of the DCP and the corresponding flow density map displayed in a 3 × 3 mm field for higher definition

**Fig. 3:**
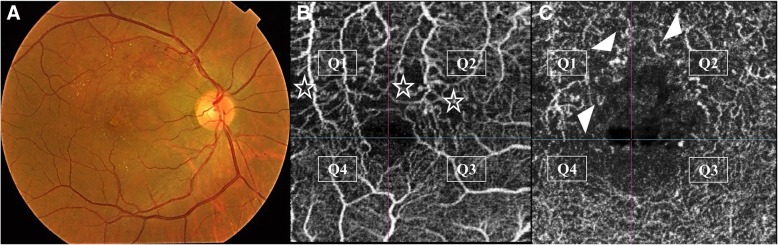
Grading of perifoveal capillary ischemia. **a**. Color fundus photo of the right eye of a 44-year old male patient with superior macular BRVO. **b**. Corresponding en-face SS-OCTA image of the perifoveal capillary arcade at the level of the SCP. A 3 × 3 mm field was selected for maximum definition. The superior portion of the perifoveal arcade shows flow-voids in quadrants Q1 and Q2 due to capillary dropout (asterisks). Note the enlargement of inter-capillary pillars, and capillary rarefaction. **c**. Corresponding en-face SS-OCTA image of the perifoveal capillary arcade at the level of the DCP. Note that the ischemic changes involving the perifoveal capillary arcade are more profound relative to the SCP image. There is extensive vascular rarefaction and diffuse flow-voids involving the superior quadrants. The FAZ is enlarged with formation of cystoid spaces in the foveal area. Scattered hyperintense streaks representing compensatory telangiectasia are seen (white arrow-heads)

En-face SS-OCTA projection of normal SCP consisted of an interwoven network of horizontal arterioles and venules connected by transverse capillaries. They both form an anastomotic perifoveal capillary circle. Arterioles are surrounded by a wider capillary-free zone compared to venules. On the other hand, normal DCP was displayed as polygonal lobules or vortices composed of capillaries converging radially on an epicenter [5]. The SCP and the DCP were evaluated in separate en-face frames for the presence of the morphological signs of ischemia previously described under FFA section. The integrated software divided each frame by horizontal and vertical crosshairs centered onto the fovea into 4 equal quadrants. In cases where the crosshairs were decentered, we resorted to manual centration on the fovea guided by the corresponding SS-OCT images. Quadrants were designated as Q1 through Q4, and were evaluated for ischemic changes. A quadrant was considered ischemic if signs of ischemia involved ≥50% of its surface area figs. 1 and 2. The perifoveal capillary arcade was considered ischemic if the continuum of the perifoveal anastomotic capillary circle was interrupted in ≥2 quadrants, i.e. affection of ≥50% of the perifoveal capillary arcade Fig. 3.

All three imaging modalities were performed on the same day. In patients with FFA images that had sufficient quality to reveal retinal perfusion, FFA was not repeated and patients underwent SS-OCT and SS-OCTA examination only. Patient selection for enrollment was undertaken by an experienced retina specialist (MM). Image grading was performed by two trained readers. Whenever discrepancy arose between the grading results of the two readers, a senior retina specialist (MM) verified the results.

### Statistical analysis

The Bivariate correlation procedure computes Pearson’s correlation coefficient. Correlation’s matrix measures how variables are related. Two variables can be perfectly related, but if the relationship is not linear, Pearson’s correlation coefficient is not an appropriate statistic. The results of “r” value were checked on “r” table to find out the significant level. Coefficient of determination (R^2^) procedure computes relative contribution of independent variable (X) in the dependent variable (Y). The Crosstabs procedure forms two-way and multiway tables and provides a variety of tests and measures of association for two-way tables. The structure of the table and whether categories are ordered determine what test or measure to use. Crosstabs’ statistics and measures of association are computed for two-way tables only. If you specify a row, a column, and a layer factor (control variable), the crosstabs procedure forms one panel of associated statistics and measures for each value of the layer factor (or a combination of values for two or more control variables).

## Results

### Patients’ characteristics

The study included 144 eyes of 138 patients (74 men and 64 women) with a mean age of 57 years (range: 22–80; SD 13). RVO was unilateral in 96% of patients. The most common type of RVO was BRVO (53%), of which 42% was major BRVO and 11% was macular BRVO. The second common type was CRVO (41%). The least common type encountered was HRVO (6%). The mean best-corrected visual acuity (BCVA) was 0.7 logMAR (range: 0.1–1.5 logMAR; SD 0.4). In terms of baseline SS-OCT findings, the mean CMT value was 385 μm (range: 146–1054 μm; SD 201), DRIL sign was detected in 82% of eyes, whereas DROL was detected in 67% of eyes. Both signs, DRIL and DROL, were displayed in 92 (64%) out of 144 eyes. FFA was performed in 74 eyes (51%). Ischemic type of RVO associated with macular ischemia was detected in 62% of eyes, which performed FFA. In terms of SS-OCTA findings, SCP layer showed ischemic changes in ≥1 quadrant in 99 eyes (69%), no ischemic changes in any quadrant in 45 eyes (31%), and perifoveal capillary ischemia in 103 eyes (71.5%). The respective flow density map showed reduced vessel density affecting ≥1 quadrant in 111 eyes (77%), and normal vessel density in 33 eyes (23%). On the other hand, the DCP layer showed ischemic changes in ≥1 quadrants in 142 eyes (99%), no ischemic changes in any quadrant in 2 eyes (1.3%), and perifoveal capillary ischemia in 137 eyes (95%). The respective flow density map showed reduced vessel density affecting ≥1 quadrants in 142 eyes (99%), and normal vessel density in 2 eyes (1.3%) Table 1.

**Table 1:** Baseline Patient Characteristics

Baseline Characteristics	N (%)
Male	74 (54)
Female	64 (46)
Age, years	
<40	15 (11)
40–50	23 (17)
51–60	45 (33)
>60	55 (40)
Baseline BCVA, logMAR	
0–0.1	5 (3)
>0.1–0.3	29 (20)
>0.3–1	82 (57)
>1	28 (19)
Type of RVO	
Major BRVO	60 (42)
Macular BRVO	16 (11)
CRVO	59 (41)
HRVO	9 (6)
SS-OCT features	
CMT, μ	
< 200	13 (9)
200–300	57 (39.5)
> 300	74 (51)
DRIL	118 (82)
DROL	96 (67)
FFA	74 (51)
Non-ischemic RVO	28 (38)
Ischemic RVO	46 (62)
SS-OCTA features	
SCP layer ischemia	
No detectable ischemic changes	45 (31)
≥1 quadrant	99 (69)
Perifoveal capillary ischemia	103 (71.5)
Normal vessel density	33 (23)
Reduced vessel density	111 (77)
DCP layer ischemia	
No detectable ischemic changes	2 (1.3)
≥1 quadrant	142 (99)
Perifoveal capillary ischemia	137 (95)
Normal vessel density	2 (1.3)
Reduced vessel density	142 (99)

*BCVA* Best-corrected visual acuity, *BRVO* Branch retinal vein occlusion, *CMT* Central macular thickness, *CRVO* Central retinal vein occlusion, *DCP* Deep capillary plexus, *DRIL* Disorganized retinal inner layers, *DROL* Disrupted retinal outer layers, *logMAR* logarithm of the minimum angle of resolution, *μ* microns, *RVO* Retinal vein occlusion, *SCP* Superficial capillary plexus, *SS-OCTA* Swept-source optical coherence tomography angiography, *SS-OCT* Swept-source optical coherence tomography

### Statistical correlation of studied anatomical parameters

#### SS-OCTA

**Fig. 4:**
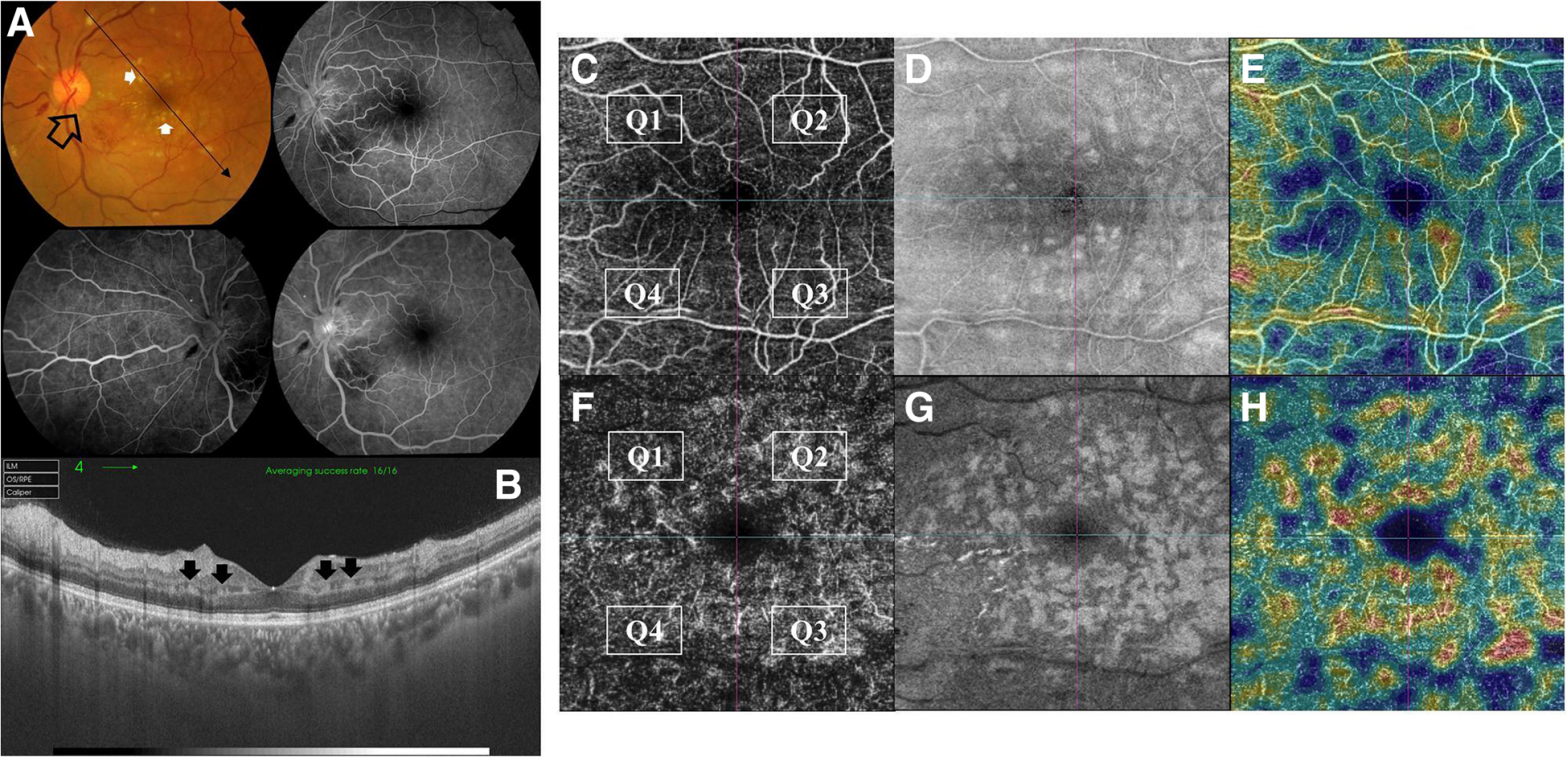
**a**. Left eye of a 41-year old male patient with inferior macular BRVO. A. Color fundus photo and FFA show the pathological arterio-venous crossing involving a tributary of the infero-temporal venous arcade (black arrow). The posterior pole shows multiple whitish rounded lesions in the para-foveal area (white arrows). The corresponding FFA shows relative hypofluorescence though no angiographic evidence of CNP, which parenthetically points to hypoperfusion of the DCP that is not delineated by FFA. Note that the FAZ is unremarkable on FFA. **b**. Corresponding SS-OCT radial scan shows multiple hyperreflective bands at the level of the INL in the para-foveal area, pathognomonic of para-central acute middle maculopathy (PAMM) lesions. **c**. Corresponding en-face SS-OCTA image in a 6 × 6 mm field. Note that the SCP and the FAZ are rather unremarkable. **d**. Corresponding en-face SS-OCT image in a 6 × 6 mm field shows the para-foveal whitish lesions seen in the color photo located at a deeper plane relative to the SCP. **e**. Corresponding flow-density map of the SCP in a 6 × 6 mm field. **f**. Corresponding en-face SS-OCTA image of the DCP. Note that all 4 quadrants show generalized vascular rarefaction and the peculiar hyperintense signal in the para-foveal area corresponding to the location of the PAMM lesions and denoting severe ischemia of the DCP. The hyperintense signal could be explained by overcrowding of vessels in the para-foveal area due to displacement by PAMM lesions or due to compensatory vasodilation secondary to ischemia. **g**. Corresponding en-face SS-OCT image at the level of the DCP in a 6 × 6 mm field. Note that the PAMM lesions with their characteristic fern-like pattern are best delineated at this plane. H. Corresponding flow-density map of the DCP in a 6 × 6 mm field

**Fig. 5:**
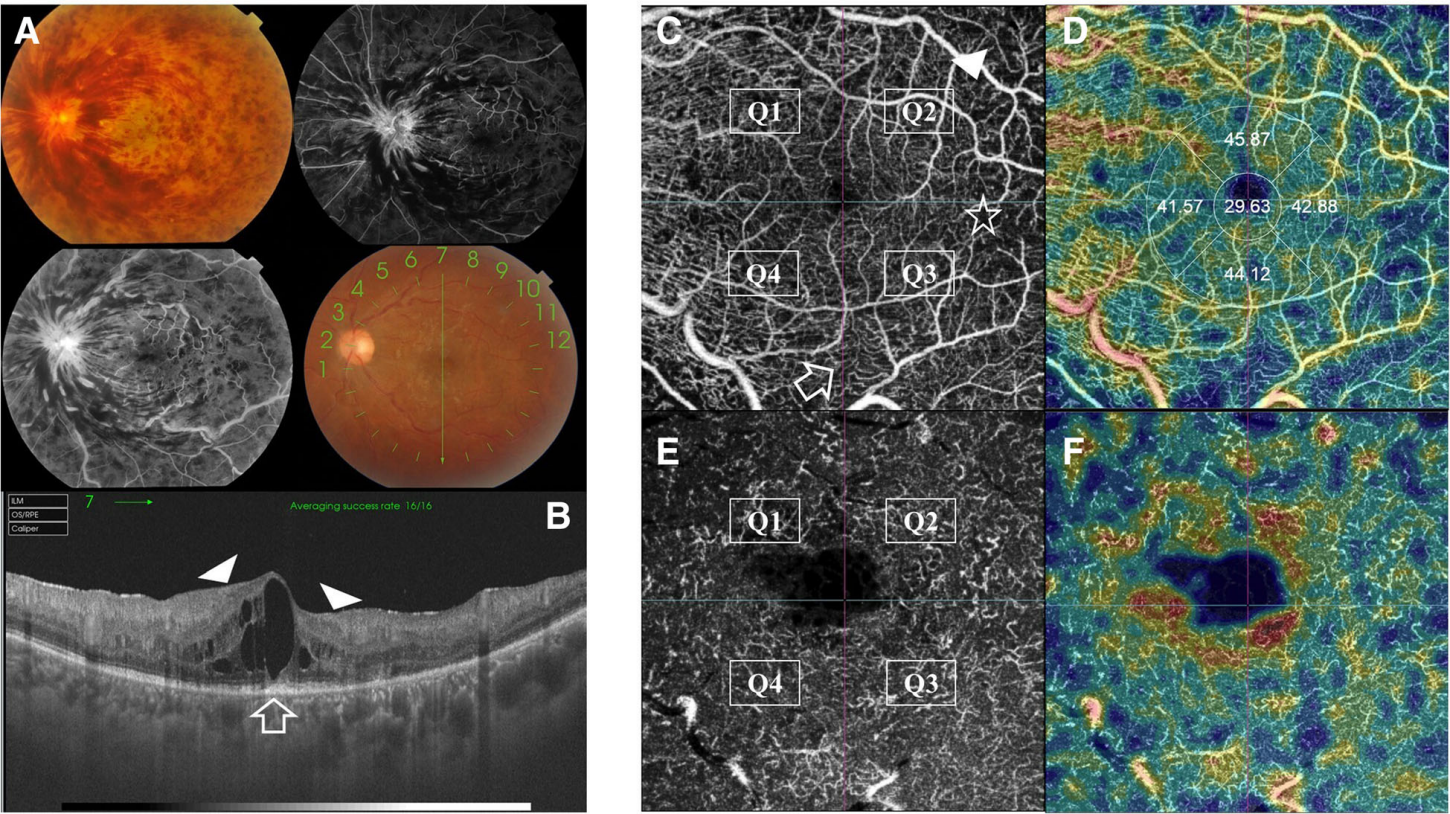
Left eye of a 51-year old male patient with ischemic type of CRVO. **a**. Color fundus photo and FFA shows the typical *blood and thunder* appearance of CRVO, with marked optic disc swelling and hyperemia along with macular edema. The lower right color photo represents the same patient after resolution of intra-retinal hemorrhages and optic disc swelling with peri-venous sheathing and CME. **b**. Corresponding SS-OCT radial scan shows a large intra-retinal hyporeflective sub-foveal space with multiple similar hyporeflective spaces in the para-foveal area indicating CME. DRIL is identified in the 1.00 mm area centered on the fovea by inability to identify the boundaries between retinal layers (white arrow-heads). Note the disrupted inner segment-outer segment (IS/OS) photoreceptors junction (white arrow). **c**. Corresponding en-face SS-OCTA image of the SCP in a 6 × 6 mm field. Note that the SCP shows only minimal ischemic changes mainly in quadrants Q2 – Q3 in the form of hypointense flow-void areas reminiscent of capillary dropout (white arrow-head), capillary pruning (asterisk) and telangiectasia (white arrow). The FAZ appearance is unremarkable. **d**, **e**. Corresponding en-face SS-OCT image and the corresponding flow-density map in a 6 × 6 mm field. **f**. Corresponding en-face SS-OCTA image of the DCP in a 6 × 6 mm field. Note almost complete *wiping-out* of the normal lobular texture of the DCP due to generalized marked vascular rarefaction in all 4 quadrants (Q1-Q4). Note the hypointense cystic spaces in the foveal area indicating CME compared to the rather normal FAZ appearance at the level of SCP. G. Corresponding en-face SS-OCT image at the level of the DCP in a 6 × 6 mm field. Note that the entire extent of cystoid spaces is best revealed at this plane. **h**. Corresponding flow-density map of the DCP in a 6 × 6 mm field

**Fig. 6:**
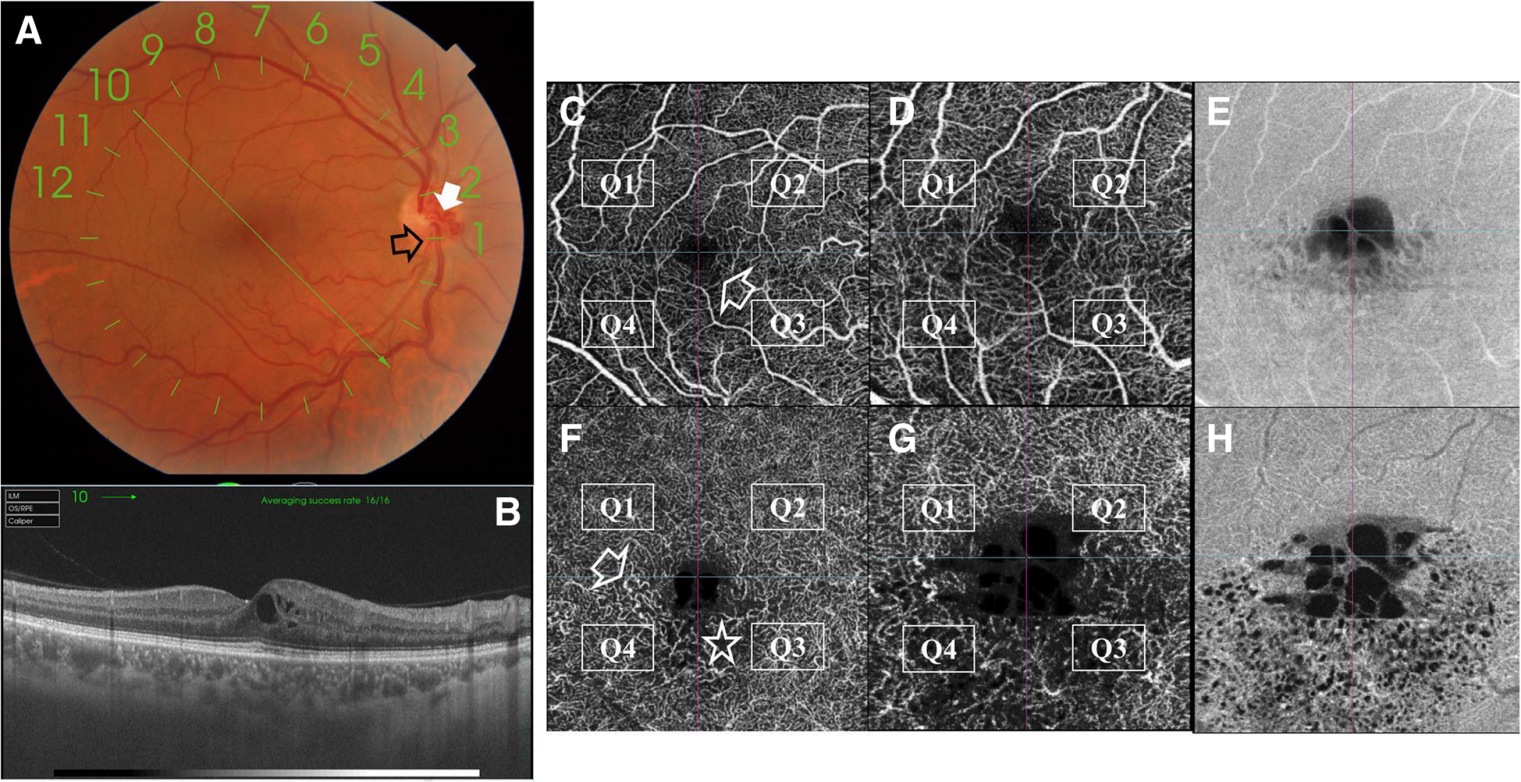
Right eye of a 48-year old male patient with longstanding inferior major BRVO. **a**. Color fundus photo showing the pathological arterio-venous crossing (black arrow). Note the collateral vessels that have developed on the optic nerve head (white arrow) reminiscent of chronic course. **b**. Corresponding SS-OCT radial scan shows multiple hyporeflective cystic space in the inferior para-foveal area. The outer retinal layers are preserved. **c**, **f**. Corresponding en-face SS-OCTA images in a 6 × 6 mm field of the SCP and the DCP, respectively. The SCP demonstrates mild ischemic changes in the form of flow-voids mainly located in the inferior quadrants Q3 and Q4. The inferior portion of the perifoveal capillary arcade is rarified (white arrow). The DCP shows flow-voids (asterisk) and compensatory telangiectasia (white arrow) in the inferior quadrants Q3 and Q4. The perifoveal capillary arcade shows pruning, and capillary loss. The FAZ is enlarged and cystoid changes are seen in the fovea. Note that the ischemic changes are more pronounced than those appreciated by examining the SCP image. **d**, **g**. En-face SS-OCTA images of the SCP and the DCP displayed in 3 × 3 mm field for higher definition. **e**, **h**. En-face SS-OCT images at the planes of the SCP and the DCP respectively, in a 3 × 3 mm field. Note that the entire extent of cystoid spaces is best revealed at the DCP plane

**Fig. 7:**
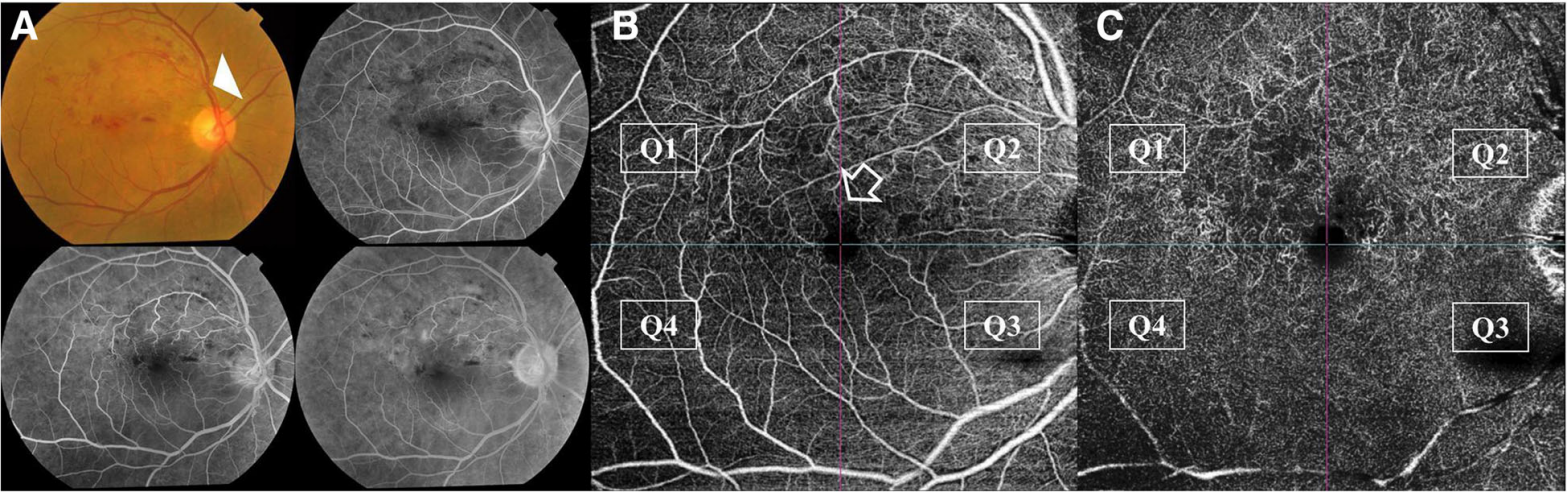
Right eye of a 50-year old female patient with superior macular BRVO. **a**. Color fundus photo and FFA. Note the pathological AV crossing affecting a macular tributary of the superior temporal venous arcade (white arrow-head). The corresponding FFA shows a non-ischemic variant of macular BRVO. **b**, **c**. Corresponding en-face SS-OCTA image in a 9 × 9 mm field of the SCP and the DCP, respectively. The SCP demonstrates mild ischemic changes in the form of flow-voids mainly located in the superior quadrants Q1 and Q2. The superior portion of the perifoveal capillary arcade is rarified (white arrow). Dilated shunt vessels straddling the horizontal raphe at the temporal edge of the perifoveal capillary arcade are seen. Note that the flow-voids in the corresponding DCP image are more extensive and often coalesce to form confluent hypointense areas denoting more diffuse capillary damage than that appreciated in the SCP image

1. Correlation between grades of ischemia in SCP versus DCP
  The SCP and the DCP did not correlate with each other in all parameters tested, which included ischemic changes per quadrant, perifoveal capillary ischemia, and vessel density per quadrant. The extent of ischemic changes was more evident in the DCP compared to the SCP, (99% in DCP versus 69% in SCP). Perifoveal capillary ischemia was more evident in the DCP compared to the SCP (95% in DCP versus 71.5% in SCP). Finally, reduced vessel density was more evident in the DCP compared to the SCP (99% in DCP versus 77% in SCP). These discrepancies were statistically significant for all parameters Table 2, figs. 4, 5 and 6.
2. Correlation between SS-OCT and SS-OCTA
  Increased CMT and presence of DROL were associated with increased severity of ischemia in DCP. The correlation was statistically significant Tables 3 and 4. The presence of DRIL did not correlate with the extent of ischemic changes in either the SCP or the DCP. However, DRIL demonstrated positive correlation with the presence of perifoveal capillary ischemia in the SCP and the DCP. The correlation was statistically significant.
3. Correlation between FFA and SS-OCTA
  Subgroup analysis for eyes which had FFA on the same day as SS-OCTA (51%) revealed that macular ischemia on FFA correlated with ischemia in the SCP layer. The correlation was statistically significant. On the other hand ischemia on FFA did not correlate with the extent of ischemia in the DCP layer Tables 5 and 6, fig. 7.

**Table 2:** SS-OCTA. Correlation matrix between SCP and DCP ischemia

	SCPQ	SPFCIsch	SVDQ	DCPQ	DPFCIsch	DVDQ
SCPQ	1	−.516(^a^)	.851(^a^)	.333(^a^)	−.109	.338(^a^)
SPFCIsch	−.516(^a^)	1	−.439(^a^)	−.108	.363(^a^)	−.135
SVDQ	.851(^a^)	−.439(^a^)	1	.410(^a^)	−.149	.448(^a^)
DCPQ	.333(^a^)	−.108	.410(^a^)	1	−.265(^b^)	.789(^a^)
DPFCIsch	−.109	.363(^a^)	−.149	−.265(^b^)	1	−.271(^b^)
DVDQ	.338(^a^)	−.135	.448(^a^)	.789(^a^)	−.271(^b^)	1

^a^Correlation is significant at the 0.01 level (2-tailed)

^b^Correlation is significant at the 0.05 level (2-tailed)

*DCPQ* Deep capillary plexus ischemia per quadrant, *DPFCIsch* Perifoveal capillary ischemia in DCP layer, *DVDQ* Vessel density per quadrant in DCP layer, *SCPQ* Superficial capillary plexus ischemia per quadrant, *SPFCIsch* Perifoveal capillary ischemia in SCP layer, *SVDQ* Vessel density per quadrant in SCP layer

**Table 3:** SS-OCTA. Correlation matrix between CMT and DCP ischemia per quadrant

CMT	DCPQ					Total
	Q0	Q1	Q2	Q3	Q4	
Total	2.7%	8.1%	35.1%	24.3%	29.7%	100.0%

Correlation coefficient = 0.246 (significant at 5%)

*CMT* Central macular thickness, *DCPQ* Deep capillary plexus ischemia per quadrant, *Q* Quadrant

**Table 4:** SS-OCTA. Correlation matrix between DROL and DCP ischemia per quadrant

		DCPQ					Total
		Q0	Q1	Q2	Q3	Q4	.00
DROL	1.00 (Present)		5.4%	24.3%	17.6%	24.3%	71.6%
	2.00 (Absent)	2.7%	2.7%	10.8%	6.8%	5.4%	28.4%
Total		2.7%	8.1%	35.1%	24.3%	29.7%	100.0%

Correlation coefficient = − 0.229 (significant at 5%)

*DCPQ* Deep capillary plexus ischemia per quadrant, *DROL* Disrupted retinal outer layers, *Q* Quadrant

**Table 5:** Correlation matrix between FFA and SCP ischemia

		SCPQ					Total
		Q0	Q1	Q2	Q3	Q4	.00
FFA (Ischemia)	1.00 (Present)	5.4%	10.8%	32.4%	4.1%	9.5%	62.2%
	2.00 (Absent)	16.2%	9.5%	9.5%	2.7%		37.8%
Total		21.6%	20.3%	41.9%	6.8%	9.5%	100.0%

Correlation coefficient = − 0.438 (significant at 1%)

*FFA* Fundus fluorescein angiography, *SCPQ* Superficial capillary plexus ischemia per quadrant, *Q* Quadrant

**Table 6:** Correlation matrix between FFA and DCP ischemia

		DCPQ					Total
		Q0	Q1	Q2	Q3	Q4	.00
FFA (Ischemia)	1.00 (Present)	1.4%	4.1%	24.3%	12.2%	20.3%	62.2%
	2.00 (Absent)	1.4%	4.1%	10.8%	12.2%	9.5%	37.8%
Total		2.7%	8.1%	35.1%	24.3%	29.7%	100.0%

Correlation coefficient = −0.044 (non-significant)

*DCPQ* Deep capillary plexus ischemia per quadrant, *FFA* Fundus fluorescein angiography, *Q* Quadrant

### Statistical correlation of SS-OCT, SS-OCTA and FFA with BCVA (logMAR)

#### Baseline BCVA versus SS-OCT

**Fig. 8:**
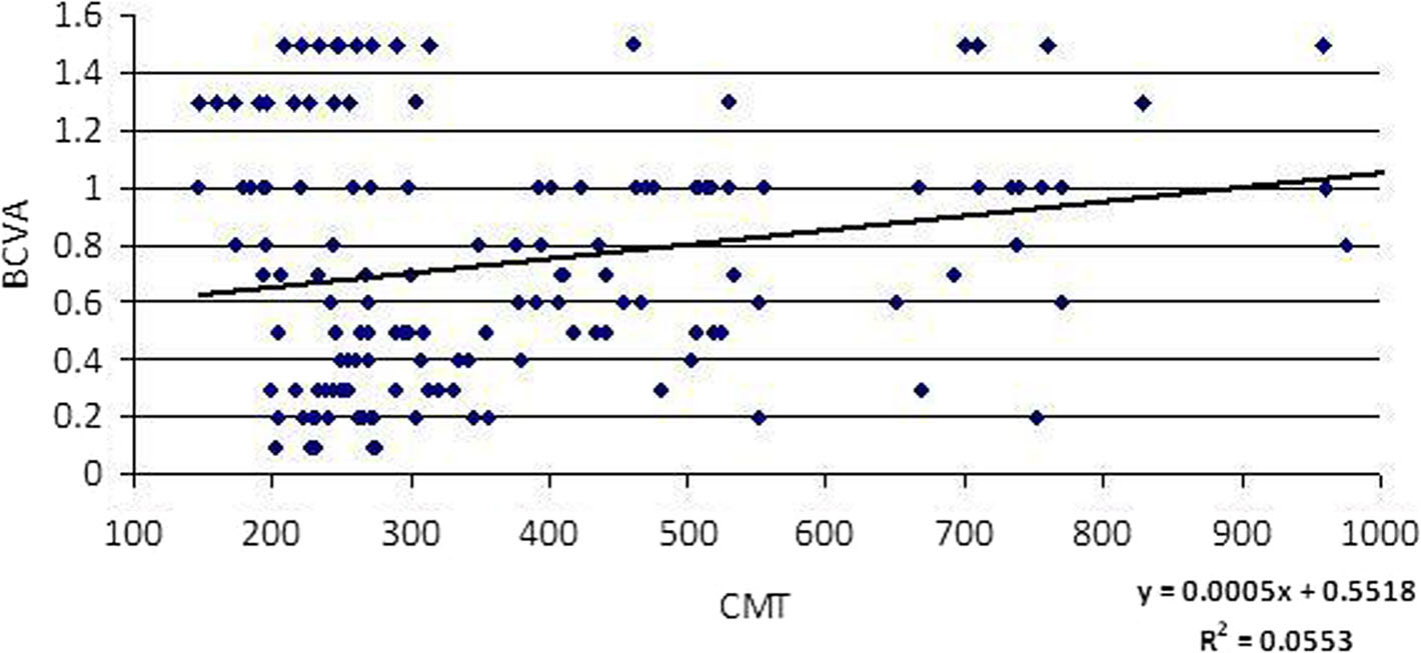
Statistical correlation between BCVA (logMAR) and CMT (μ). BCVA, Best-corrected visual acuity; CMT; Central macular thickness

Increased CMT, DROL and DRIL were significant contributing factors in diminished visual acuity. Table 7, fig. 8.

**Table 7:** Correlation and relative contribution between BCVA and SS-OCT features (CMT – DRIL – DROL)

BCVA (logMAR)	Correlation (R)	Relative Contribution (R^2^)	Significance
CMT	0.235	5.52	^b^
DRIL	−0.180	3.24	^a^
DROL	−0.179	3.20	^a^

(^a^) Significant

(^b^) Highly significant

*BCVA* Best-corrected visual acuity, *CMT* Central macular thickness, *DRIL* Disorganized retinal inner layers, *DROL* Disrupted retinal outer layers, *logMAR* logarithm of the minimum angle of resolution

#### Baseline BCVA versus SS-OCTA

Poor baseline BCVA correlated with increased severity of ischemia in the SCP and the DCP layers. The correlation was statistically significant. In other terms, the severity of ischemia was a significant contributing factor to poor baseline BCVA. Table 8.

**Table 8:** Correlation and relative contribution between BCVA and SS-OCTA features

BCVA (logMAR)	Correlation (R)	Relative Contribution (R^2^)	Significance
SCPQ	0.296	8.76	^b^
SPFCIsch	−0.126	1.59	NS
SVDQ	0.301	9.06	^b^
DCPQ	0.193	3.72	^a^
DPFCIsch	−0.092	0.85	NS
DVDQ	0.275	7.56	NS

(*NS*) Non-significant

(^a^) Significant

(^b^) Highly significant

*BCVA* Best-corrected visual acuity, *DCPQ* Deep capillary plexus ischemia per quadrant, *DPFCIsch* Perifoveal capillary ischemia in DCP layer, *DVDQ* Vessel density per quadrant in DCP layer, *logMAR* logarithm of the minimum angle of resolution, *SCPQ* Superficial capillary plexus ischemia per quadrant, *SPFCIsch* Perifoveal capillary ischemia in SCP layer, *SVDQ* Vessel density per quadrant in SCP layer

#### Baseline BCVA versus FFA

Subgroup analysis for eyes which had FFA on the same day as BCVA assessment (51%) revealed that macular edema and status of macular perfusion on FFA did not correlate significantly with BCVA. Table 9.

**Table 9:** Correlation and relative contribution between BCVA and FFA findings

BCVA (logMAR)	Correlation (R)	Relative Contribution (R^2^)	Significance
FFA (Ischemia)	−0.127	1.613	NS
FFA (Macular edema)	−0.010	0.01	NS

*BCVA* Best-corrected visual acuity, *FFA* fundus fluorescein angiography, *logMAR* logarithm of the minimum angle of resolution, *NS* Non-significant

## Discussion

Much of our understanding on the relationship between visual function and macular ischemia in patients with RVO is derived from FFA. The conclusion that visual dysfunction is in lockstep with ischemic changes of the FAZ as reflected by FFA is at most a conjecture, with discrepant reports describing the relationship between the grade of visual dysfunction and status of the FAZ following RVO [25–27]. The reason is that FFA, due to its inherent limitations, fails to include critical information on structural changes of the retinal micro-structure and of the DCP, which are cardinal determinants of the visual outcome in patients with RVO [28–31]. Accordingly, absolute reliance on FFA for evaluation of macular ischemia following RVO episode could pose major compromise of our perception of the perfusion profile of the macula. In the present study, we focused on quantifying the pathological changes in the retinal vascular plexuses secondary to RVO using SS-OCTA and to devise a perfusion profile of the macula that would accurately represent the ischemic insult inflicted. Furthermore, we correlated our findings with the anatomic changes evident on SS-OCT and FFA and their relation to the visual function. Our results showed a statistically significant discrepancy between the grade of ischemia in SCP and DCP on SS-OCTA, in the sense that more ischemic quadrants were present at the level of the DCP relative to the SCP, which showed less ischemic damage. Moreover, perifoveal capillary ischemia and reduced vessel density per quadrant were more pronounced in the DCP relative to the SCP. These results confirm reports from several authors that ischemic damage in RVO is preferentially main-seated in the DCP and often precedes SCP ischemia [4, 7, 10, 29–33]. In comparison with our results, Coscas et al. [6] reported in a series of 54 patients with RVO, more extensive ischemic damage in the DCP relative to the SCP (84.3% vs. 58.8%, respectively). Cardoso et al. [13], demonstrated in a reliability analysis of 81 eyes with RVO that capillary changes associated with ischemia were primarily located in the DCP. Chung et al. [34], reported in a study recruiting 12 patients with RVO, patchy CNP areas involving the DCP that were not detected on the SCP using OCTA. Further corroborating evidence of more selective DCP affection in RVO was demonstrated in a retrospective series including 17 eyes by Samara et al. [8], and in two series by Suzuki et al. [35, 36] that included 12 eyes with BRVO and CRVO and 28 eyes with BRVO, respectively. These studies detected abnormally enlarged FAZ that was more pronounced in the DCP in eyes with RVO compared to the fellow unaffected eye. Moreover, Suzuki et al. [35], detected reduced retinal flow area that was most marked in the DCP in eyes with RVO compared to the unaffected fellow eye. Similarly, Adhi et al. [9], reported in a prospective case series of 23 patients with RVO, decreased vascular perfusion in the DCP that surpassed in frequency of occurrence and severity of involvement that of the SCP. Another important aspect of the present study, is the analysis of the correlation between SS-OCT anatomical features with ischemic changes revealed on SS-OCTA. Increased CMT and DROL were the most sensitive SS-OCT indices for DCP ischemia. On the other hand, DRIL was the least reliable SS-OCT index for SCP or DCP, though it had a direct proportional correlation with the severity of perifoveal capillary ischemia. Henceforth, we could propose a high-risk SS-OCT profile that includes increased CMT, DROL and DRIL and that portends significant ischemia in the DCP and in the perifoveal capillary arcade, respectively. Our conclusion on the positive correlation between increased CMT and DCP ischemia is ratified by Spaide’s theory on the cause-effect relationship between compromised blood flow within the DCP and reduced clearance of interstitial fluid from the retina and that eventually leads to macular edema [37]. Further corroborating evidence is provided from the work of Tsuboi et al. [38], who demonstrated a statistically significant positive correlation between flow-voids; equivalent to diminished vessel density in the SCP and the DCP and persistent macular edema in BRVO patients. In comparison, our results revealed that FFA was not reliable in assessment of ischemia. FFA representation of macular perfusion was significantly proportionate to SCP perfusion on SS-OCTA but in most cases it could not reveal the entire extent of macular ischemia as revealed by SS-OCTA depiction of DCP. Our findings are in line with two studies of the retinal vascular layers by Mendis et al. [39], and Spaide et al. [10], who compared FFA versus confocal laser scanning microscopy, and FFA versus OCTA respectively in evaluating the retinal vascular layers. Both studies concluded that FFA was unable to provide complete information on the DCP. In accordance with our findings, Chung et al. [34], reported poor agreement between FFA and OCTA in revealing CNP following RVO with clear superiority of OCTA over FFA (91.67% vs. 58.33%), particularly in DCP non-perfusion and in detection of perifoveal capillary ischemia. Similarly, two case series including 28 eyes and 10 eyes with BRVO by Suzuki et al. [36], and Rispoli et al. [40], respectively, and one case report of ischemic BRVO by Kuehlewein et al. [41], found that OCTA was superior to FFA in detection of CNP areas and in delineating them with higher-resolution, and in detection of microvascular abnormalities and FAZ dimensions. It is worthy of note that the latter authors used the SS-OCTA technology in their study. In terms of versatility of the studied imaging modalities in predicting visual outcome after RVO, our findings revealed that increased CMT, DRIL, and DROL on SS-OCT, and SCP and DCP ischemia on SS-OCTA were significant predictors of poor visual outcome; whereas FFA features that included macular edema and macular ischemia did not correlate with the visual outcome of those patients. These findings are in line with Wakabayashi et al. [7], who studied the correlation between OCTA and BCVA in a series of 85 eyes with BRVO. These authors found that DCP ischemia is the most significant determinant of BCVA. Furthermore, the authors reported significant correlation between DCP ischemia on OCTA and worsening macular edema and disrupted photoreceptors layer on OCT. Our results are congruous with those of Samara et al. [8], who detected that logMAR BCVA correlated positively with foveal thickness on OCT, and negatively with vascular density in DCP on OCTA. The present study has several confounding factors, of which the most salient is its retrospective design, which allowed entry of patients with important epidemiologic and phenotypic variation of RVO. This uneven stratification might have posed an information bias that could affect our interpretation of the correlation between the tested parameters in more than one aspect. Firstly, almost 50% of recruited patients had CRVO and HRVO, which implicate more extensive DCP ischemia on SS-OCTA, more diffuse structural changes on SS-OCT and worse impact on visual acuity than BRVO. Secondly, recruited patients had different stages of RVO whether acute or chronic. Accordingly, those patients were going through different stages of microvascular remodeling following the retinal venous occlusive event with fairly wide-variation in time course and quantity. Thirdly, patients were allowed to enroll in the study regardless of whether they were treatment-naïve or had received single or multiple lines of therapy, which could have important impact on the vessel density in the SCP and DCP, and on the structural changes on SS-OCT. Finally, the current study lacked concurrent evaluation of the perfusion of the peripheral retina, which has an important impact on macular edema. Rather the study focused on grading the perfusion in the macular area and the posterior pole. A more comprehensive evaluation of RVO would include correlation between the current findings and the grade of non-perfusion in the retinal periphery using wide-field OCTA or a montage of multiple 9 × 9 mm or 12 × 12 mm scans.

## Conclusion

The present study was able to affirm the efficacy of SS-OCTA in qualitative assessment of macular perfusion status and its ability to perform differential layer segmentation of the retinal vascular plexuses to define the extent and location of maximum ischemic insult following RVO, hence endorsing its role as more representative grader of ischemic damage in the posterior pole than conventional FFA. In addition, we were able to correlate the anatomic features on SS-OCT, vascular perfusion on SS-OCTA and visual function and to identify significant predictors on SS-OCT and SS-OCTA for visual outcome after RVO.

## Data Availability

The statistical data used to support the findings of this study are included within the article. The data collected from history taking and clinical examination of patients recruited in the current study are confidential. Access to these data is restricted by MEDIC Eye Center, Tanta, Egypt in accordance with the patients’ data protection policy. Data are available for researchers who meet the criteria for access to confidential data through contacting MEDIC Eye Center director, Professor Magdy Moussa.

## Abbreviations

anti-VEGF: Anti-vascular endothelial growth factor
BCVA: Best-corrected visual acuity
BRVO: Branch retinal vein occlusion
CME: Cystoid macular edema
CMT: Central macular thickness
CNP: Capillary non-perfusion
CRVO: Central retinal vein occlusion
DCP: Deep capillary plexus
DD: Disc diameter
DRIL: Disorganized retinal inner layers
FAZ: Foveal avascular zone
FFA: Fundus fluorescein angiography
HRVO: Hemi-retinal vein occlusion
INL: Inner nuclear layer
IPL: Inner plexiform layer
IS/OS: Inner segment – outer segment
IVTA: Intravitreal triamcinolone acetonide
logMAR: Logarithm of the minimum angle of resolution
OCT: Optical coherence tomography
OCTA: Optical coherence tomography angiography
OCTARA: Optical coherence tomography angiography ratio analysis
OPL: Outer plexiform layer
RVO: Retinal vein occlusion
SCP: Superficial capillary plexus
SS-OCT: Swept-source optical coherence tomography
SS-OCTA: Swept-source optical coherence tomography angiography
